# Supplementary material for: Decoding visual colour from scalp electroencephalography measurements
Source: Neuroimage. 2021 Aug 15;237:118030. doi: 10.1016/j.neuroimage.2021.118030 (PMC8285579; doi:10.1016/j.neuroimage.2021.118030)
Supplement: Supplementary file 1 [file mmc1.docx]

## **Supplementary materials**

During the delay period, participants were presented with a bilateral distractor for 100 ms which consisted of two coloured discs (of the same colour) on half of the trials or two oriented Gabor gratings (of the same orientation) on the other half. Sixteen evenly spaced colour and orientation features were defined for the distractor, which were binned into 8 bins for the following decoding analyses to ensure a similar number of trials per feature. We epoched the data from 400 ms prior to distractor onset to 800 ms post distractor onset. The same spatial-temporal decoding methods described in the manuscript were applied to the neural data. Classifier evidence for colours was an order of magnitude higher than that observed for items in the encoding frame. This may be due to the fact that the distractors only contained a single feature that was identical for the left and right side.


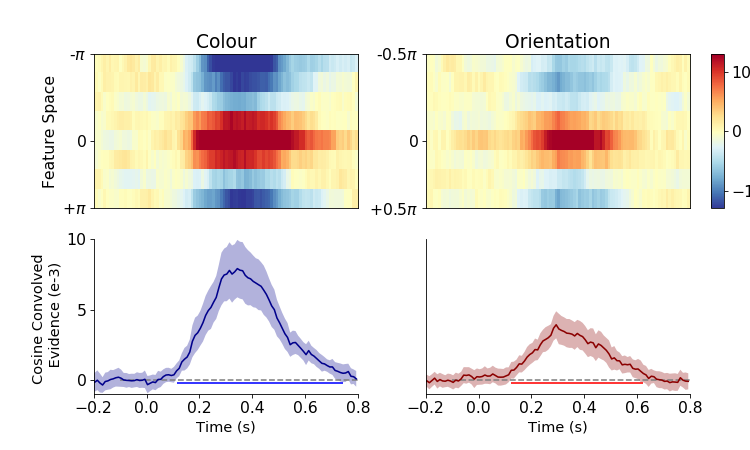


**A)**

**B)**


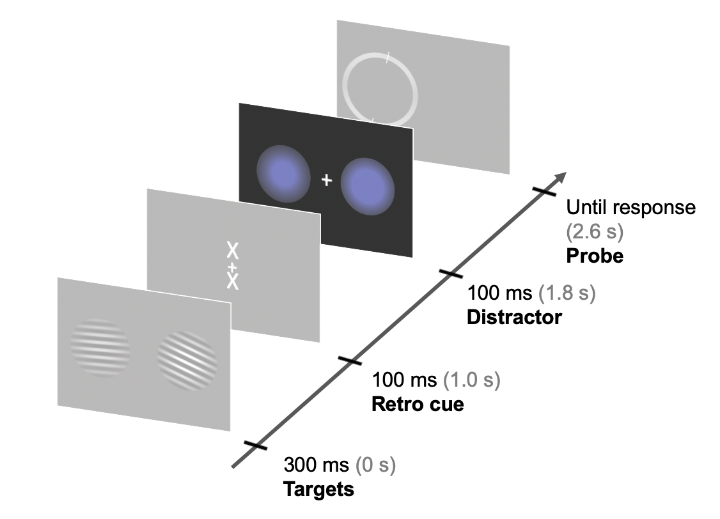


**Fig. S1. Decoding the colour and orientation of the visual distractors.** A) On every trial, participants were presented with a bilateral array of distractors that consisted of a single feature. On a randomly selected half of trials this was a colour and on the other half of trials this was an orientation. The decoding analyses for the distractor epoch were carried out in the same way as in the encoding epoch, colour and orientation features were binned into 8 stimulus bins and decoding analyses were applied in an identical fashion for the distractor epoch. B) Mean cosine-convolved evidence with red lines for colour, blue for orientation. Two-dimensional, decoding tuning curves with the likelihood for target colour or orientation (value “0”) and all neighbouring values of theta. Error bars show 95% confidence intervals, calculated across participants (n = 30).

When applying LDA to the data from the 17 posterior electrodes for each time point, we do not reduce the dimensionality using PCA. Other preprocessing steps are identical. Single time-point LDA decoding shows that colour decoding was first significant after 125 ms, until 595 ms (**Fig. S2**). Orientation decoding was only significant after 185 ms, until 625 ms.


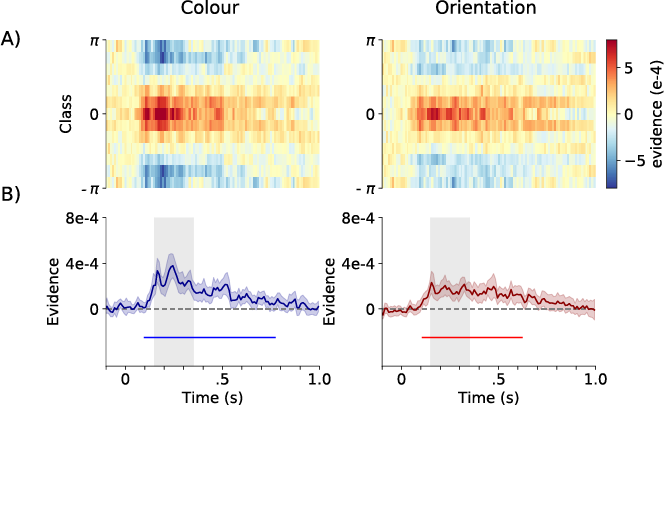


**Fig. S2. Decoding performance for colours and orientations with single time-point decoding.** A) Two dimensional tuning curves with the likelihood for target colour or orientation and all neighbouring values of theta. B) Mean cosine-convolved evidence with red lines for colour, blue for orientation. Cluster-permutation corrected significant time points are indicated with horizontal lines. Grey-shaded area between 150 to 350 ms is used for subsequent analyses. Error bars show 95% confidence intervals.

**
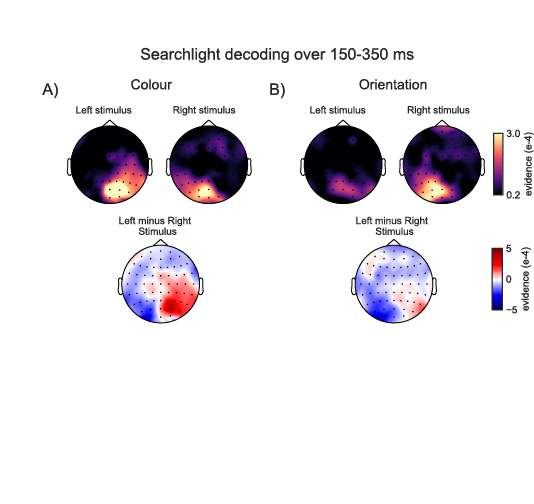
**

**Fig. S3. Colour and orientation decoding are primarily driven by posterior electrodes contralateral to the decoded stimulus.** After averaging over data within the 150- to 350-ms time window, we ran a searchlight analysis using only the spatial pattern (in contrast to **Fig. 3** where we also incorporated spatial-temporal information). Top row topographies show which electrodes show highest mean cosine-convolved evidence for A) colours and B) orientations presented on the left and on the right. Posterior contralateral electrodes show highest evidence. Bottom panels show the difference between left and right feature decoding topographies, highlighting the lateralisation of the evidence.

Instead of concatenating data over the window of 150 ms until 350 ms post stimulus onset, we averaged data into an average pattern and proceeded with the same analyses as depicted in (**Fig. 4**). For colour bins, 11/12 colours could be decoded significantly against a *p* < .05 threshold (**Fig. S4**; mean t_29_ = 3.391; min = 0.530; max = 6.040), and for orientation all 6/12 orientations could be decoded significantly against an uncorrected single-tail threshold (mean t_29_ = 2.436; min = 0.530; max = 4.561). After applying Bonferroni correction, 5/12 colour bins were significant compared to 4/12 orientations.

**
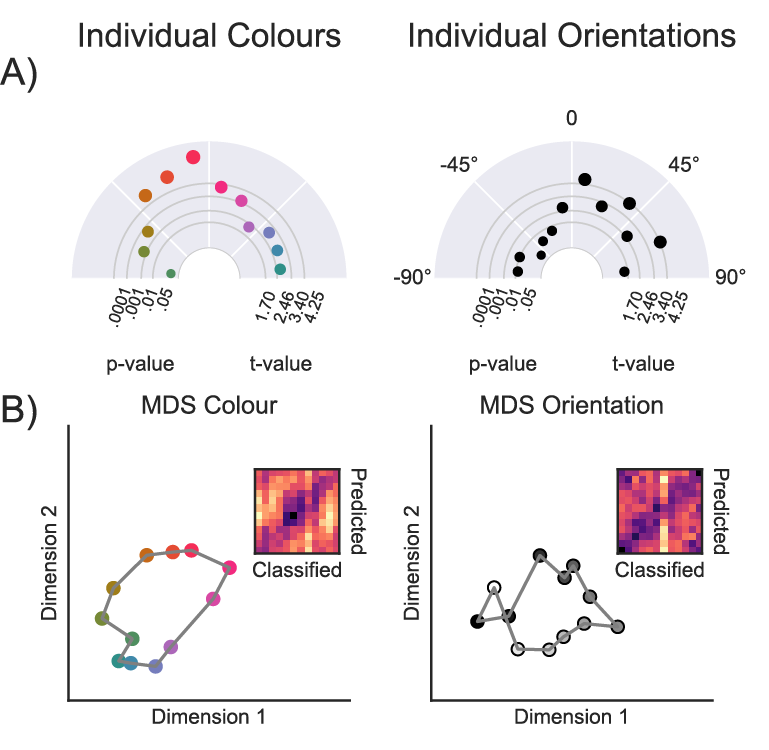
**

**Fig. S4. Decoding of individual features between 150 and 350 ms.** A) This polar plot illustrates the decoding evidence of 12 individual colours and orientations. The distance from the centre depicts the t-value of testing the cosine-convolved tuning curve for a single feature, left and right combined, across participants, relative to zero. The size of the dots mirrors the magnitude of the evidence for that feature B) Two-dimensional visualisation of the similarity matrix in which we observed a circular configuration for colour features. Dissimilarity matrices show feature (colour or orientation) on the y-axis and distance between bins of the tuning curve on the x-axis.


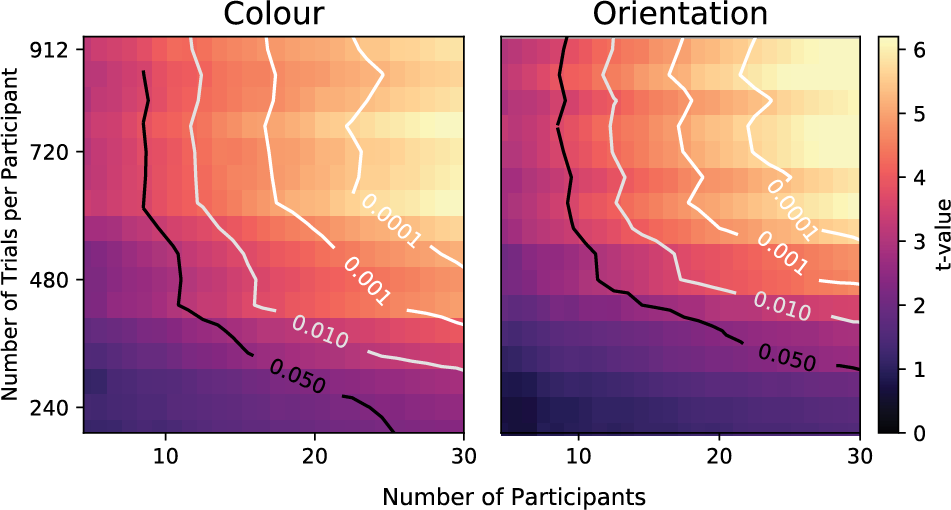


**Fig. S5. Feature decoding as a function of trial and participant numbers.** Average cosine-convolved evidence between 150 and 350 ms for the average of left and right feature decoding. Contours mark the .05, .01, .001, and .0001 p-value thresholds for n-1 degrees of freedom.

To investigate the contribution of alpha-band power to our current results, data were bandpass filtered between 8 – 12 Hz and submitted to a Hilbert transform to compute the time-resolved magnitude of the complex analytical signal. The resulting alpha-power estimation was passed through the same classification procedure described in the methods section of the manuscript. While we did find one relatively weak significant cluster in the single-timepoint decoding data relative to stimulus encoding (385 – 999 ms; **Fig. S6B**, left), this effect occurred much later than the decoding from the broadband signal. Moreover, unlike the broadband decoding results, this effect did not replicate across the other three analyses depicted in **Fig. S6**.

A)

B)


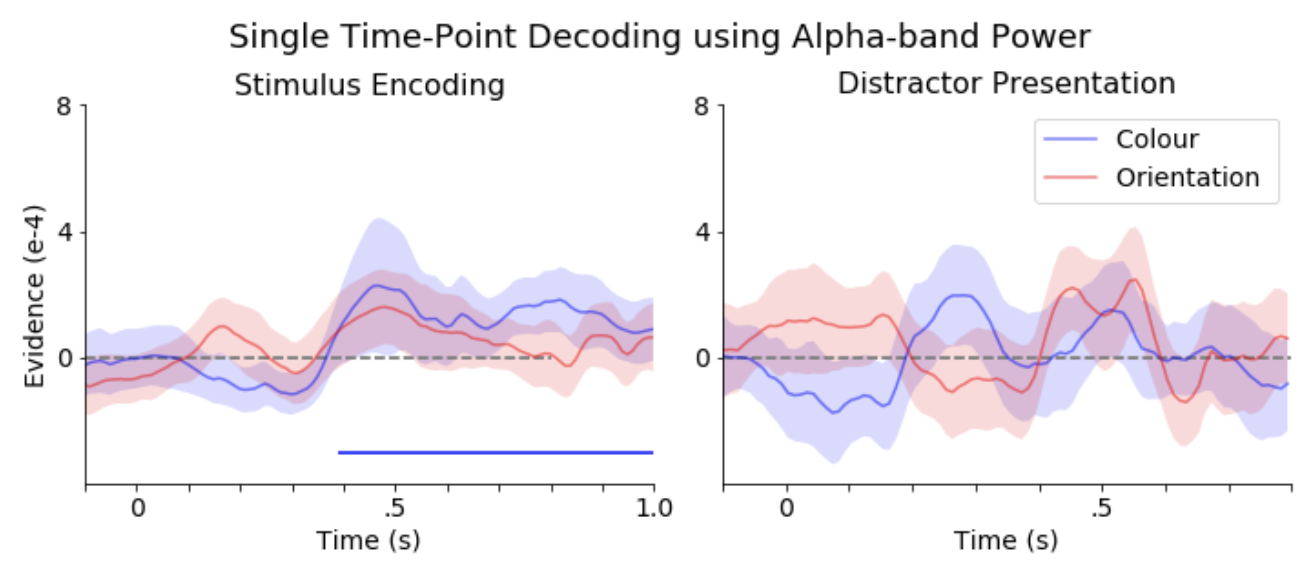

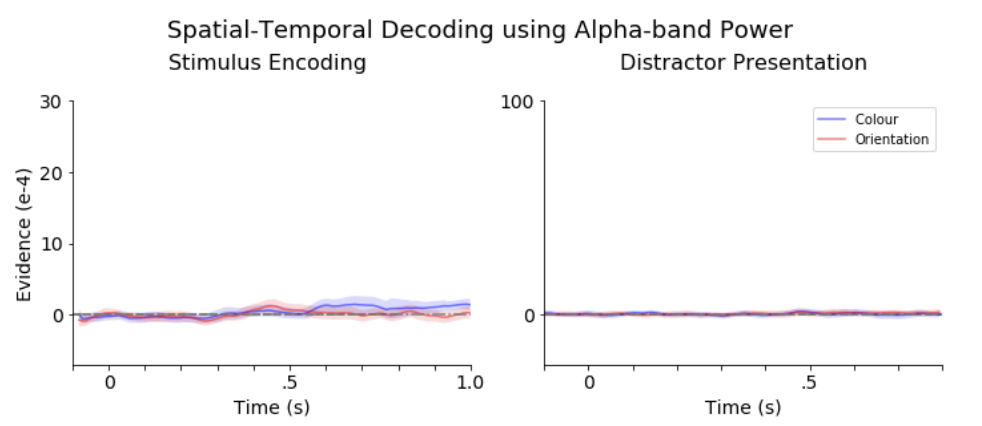


**Fig. S6. LDA classification of colour and orientation using Alpha-band power in the stimulus encoding and distractor presentation epoch.** A) Cosine convolved evidence in spatial-temporal LDA following stimulus presentation for colour and orientation, depicted in blue and red, respectively. The left panel shows evidence during the stimulus encoding interval and the right panel for during the distractor presentation interval. B) Analyses using Alpha-band power were repeated with single time-point classification technique (**Fig. S2-5**), for both the stimulus and distractor intervals. Y-axis limits reflect the same scale as the respective stimulus-decoding plots (**Fig. 2, Fig S1, Fig. S2**). Error bars indicate 95% confidence intervals.
